# Simultaneous dual-isotope SPECT imaging using a cardiac cadmium-zinc-telluride camera with various Technetium-99 m to Iodine-123 ratios

**DOI:** 10.1186/s41824-025-00272-6

**Published:** 2025-10-20

**Authors:** Takayuki Shibutani, Masahisa Onoguchi, Hiroto Yoneyama, Takahiro Konishi, Kenichi Nakajima

**Affiliations:** 1 Department of Quantum Medical Technology, Institute of Medical, Pharmaceutical and Health Sciences, Kanazawa University, 5-11-80, Kodatsuno, Kanazawa, Ishikawa Japan; 2 Department of Radiological Technology, Kanazawa University Hospital, Kanazawa, Ishikawa Japan; 3 Department of Nuclear Medicine, Kanazawa University, Kanazawa, Ishikawa Japan

**Keywords:** Simultaneous dual-isotope, Gaussian filter, Myocardial SPECT, Scatter correction

## Abstract

**Objective:**

Cadmium-zinc-telluride (CZT) SPECT cameras offer high energy resolution. This allows simultaneous dual-isotope (SDI) acquisition even when ^123^I and ^99m^Tc photon peaks are close. This study aimed to reveal the impact of image quality for ^99m^Tc/^123^I ratios on SDI images.

**Methods:**

We created normal and inferior wall defect myocardial models using an anthropomorphic myocardial phantom. The doses of ^99m^Tc/^123^I ratios were set to 1.0, 3.5, 5.0 and 6.5 (referred to as Tc/I_1.0, Tc/I_3.5, Tc/I_5.0 and Tc/I_6.5 conditions). We acquired SPECT images using D-SPECT Cardio. The acquisition time was adjusted to achieve 1.5 × 10^6^ left ventricular ^123^I counts as a clinical reference. Short-axis images were reconstructed with (SC) and without (NC) scatter correction. Kernel and Gaussian-standard values were set to 1 and 1.0 as default conditions. The filter parameters were changed to 1, 3 and 5 for the kernel settings, and 0.25–1.0 for Gaussian standards. The image quality of normal and defective myocardia at different ^99m^Tc/^123^I ratios was evaluated as cavity contrast on short-axis images, the percent coefficient of variance (%CV) and %uptake on polar maps. In addition, these reconstruction conditions were applied to a patient with acute coronary syndrome, supporting the phantom-based findings.

**Results:**

In the normal myocardial phantom, %uptake in the inferior and inferolateral myocardial walls was significantly lower in SC images than in NC images for both ^99m^Tc and ^123^I. In SC images, ^99m^Tc counts in the inferoseptal myocardial wall slightly decreased along with the ^99m^Tc/^123^I ratio, whereas ^123^I counts in the inferolateral walls declined as the ^99m^Tc/^123^I ratio increased to 6.5. Higher kernel and Gaussian standard settings resulted in lower %CV values in both NC and SC ^99m^Tc and ^123^I images, improving image uniformity. Overall, NC images showed more homogeneous myocardial distribution in the inferior wall compared with SC images.

**Conclusion:**

Image quality and correction effects are influenced by ^99m^Tc/^123^I ratios, resulting in heterogeneity in the inferior wall of the normal myocardium due to SC. Adjusting kernel parameters of the Gaussian filter or using NC images may help improve image quality when ^99m^Tc/^123^I ratios are very high or low.

## Introduction

Myocardial single photon emission computed tomography (SPECT) using simultaneous dual-isotope (SDI) image acquisition is a valuable tool for diagnosing cardiovascular diseases. It can identify culprit coronary arteries, predict viability in patients with acute coronary syndrome (ACS), detect stunned myocardia, and assess ischemia in patients when stress tests are not indicated (Sato et al. 2008; Kobayashi et al. 1998; Nakajima et al. 1995; Nakahara et al. 2001). Myocardial SPECT with SDI has used radiopharmaceuticals labeled with Thallium-201 (^201^Tl) and Iodine-123 (^123^I) in conventional NaI (Tl) scintillator-type SPECT scanners due to their energy characteristics. However, the downscatter from ^123^I into the ^201^Tl energy spectrum, as well as crosstalk between the 167 and 159 keV gamma rays of ^201^Tl and ^123^I, respectively, have been identified as technical pitfalls that degrade image quality (Yang et al. 1997; Nakajima et al. 1990). Despite these limitations, SDI myocardial SPECT is widely applied clinically, as scatter correction (SC) and other methods of crosstalk mitigation have been implemented to minimize the impact of downscatter and crosstalk (Saitoh et al. 1995; Nishimura et al. 1994).

Cadmium-zinc-telluride (CZT) SPECT cameras offer high energy resolution, which allows SDI acquisition of radionuclides with close photon peaks such as ^99m^Tc combined with ^123^I (Niimi et al. 2018). Sensitive CZT SPECT also facilitates low-dose or short-time acquisition (Slomka et al. 2019). The technical and clinical research of SDI image acquisition using cardiac CZT SPECT with ^99m^Tc and ^123^I is being conducted (Niimi et al. 2018; Assante et al. 2022; Yamada et al. 2021; Blaire et al. 2016, 2018; Takahashi et al. 2015). Clinical studies have used doses of ^99m^Tc/^123^I ratios such as 185/74 (Slomka et al. 2019) and 600/111 (Assante et al. 2022) MBq. The ^99m^Tc/^123^I ratio has similarly been varied to assess the quality of myocardial CZT SPECT phantom images, with reported values of 1.0 (Niimi et al. 2018; Takahashi et al. 2015) and 2.0 (Blaire et al. 2016, 2018). Such different doses and concentrations in clinical and phantom studies using SDI myocardial SPECT with a CZT detector might influence image quality due to ^99m^Tc and ^123^I crosstalk and downscatter from ^123^I, particularly affecting the heterogeneity of normal myocardium and the reducing image contrast (Fan et al. 2015; Velo et al. 2023). However, the impact of varying ^99m^Tc/^123^I ratios on image quality in CZT detector-based SDI myocardial SPECT has not been systematically evaluated. This study aimed to reveal image quality and correction effects at various ^99m^Tc/^123^I ratios in CZT SPECT.

## Materials and methods

**Fig. 1:**
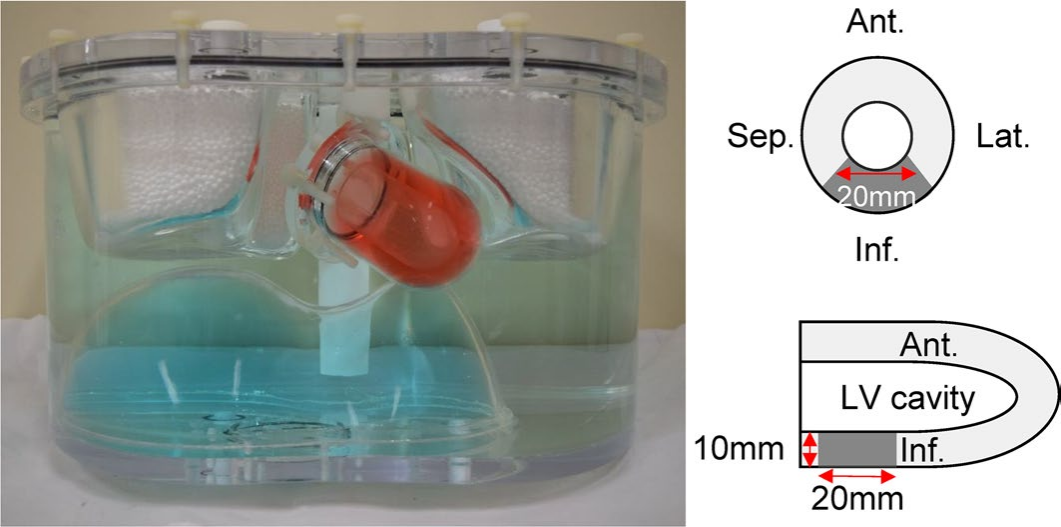
Phantom structure of anthropomorphic myocardial phantom. Ant, anterior; Inf, inferior; Lat, lateral; LV, left ventricle; Sep, septal

We created normal and inferior wall defect myocardial models using an anthropomorphic myocardial phantom (Fig. 1). The ^99m^Tc/^123^I ratios of injected doses were 1.0, 3.5, 5.0 and 6.5 (referred to as Tc/I_1.0, Tc/I_3.5, Tc/I_5.0 and Tc/I_6.5), which were determined according to possible clinical settings of ^123^I-meta-iodobenzylguanidine (MIBG) or ^123^I- beta-methyl-p-iodophenyl-pentadecanoic acid (BMIPP) and ^99m^Tc-labeled myocardial perfusion imaging (Niimi et al. 2018; Assante et al. 2022; Yamada et al. 2021; Blaire et al. 2016, 2018; Takahashi et al. 2015). Myocardial uptake relative to injected doses was 1.5% and 5.4% for ^99m^Tc and ^123^I as described (Kubo et al. 1992; Torizuka et al. 1991). The resulting radioactive concentrations of ^99m^Tc in the myocardium were 14, 49, 69, and 90 kBq/mL for Tc/I_1.0, Tc/I_3.5, Tc/I_5.0, and Tc/I_6.5, respectively. The radioactive concentration of ^123^I was maintained at 50 kBq/mL across all ^99m^Tc/^123^I ratio. This yielded myocardial radioactive concentrations for ^99m^Tc/^123^I ratios of approximately 0.3, 1.0, 1.4 and 1.8, respectively. The radioactive concentration ratio in the myocardium, liver and mediastinum were set at 14, 8 and 1, respectively for each ^99m^Tc/^123^I ratio in the phantom. The left ventricular (LV) chamber contained nonradioactive water.

**Fig. 2:**
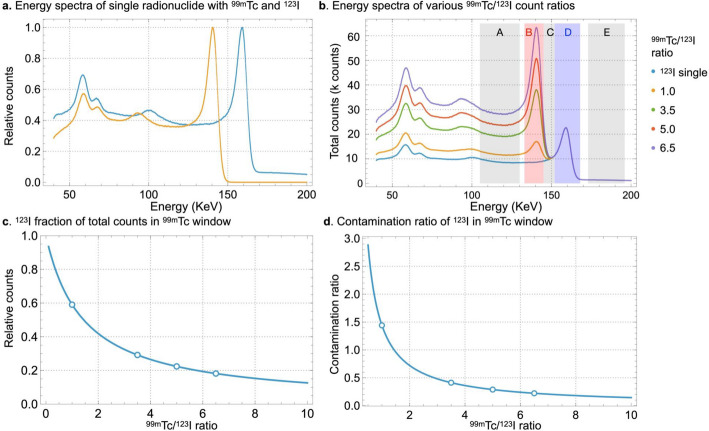
Energy spectrum of simultaneous dual-isotope acquisition using ^99m^Tc and ^123^I. Windows: A, ^99m^Tc scatter (105‒130 keV); B, ^99m^Tc main (133‒145 keV); C, ^123^I scatter (145‒152 keV); D, ^123^I main (152‒168 keV); E, ^123^I upper (173‒196 keV) windows. Panels (**a**) Normalized energy spectra from single radionuclide acquisition with ^99m^Tc and ^123^I; (**b)**, Composite energy spectra for various ^99m^Tc and ^123^I count ratios based on the phantom measurements. The agreement between these composite spectra and directly acquired mixed-radionuclide data was confirmed; (**c)**, ^123^I fraction of the total counts in the ^99m^Tc window; panel **d**, ^123^I-to-^99m^Tc contamination ratios within the ^99m^Tc window. Open circles in panel **c** and **d** denote the ^99m^Tc/^123^I ratios of 1.0, 3.5, 5.0 and 6.5

We acquired images using D-SPECT Cardio with a wide-angle parallel-hole collimator (Spectrum Dynamics Medical Ltd., Caesarea, Israel). The acquisition matrix were 16 × 64 with a pixel size of 4.92 mm, and the scan time was adjusted to achieve 1.5 × 10^6^ LV ^123^I counts as a clinical reference. Figure 2a shows normalized ^99m^Tc and ^123^I energy spectra (peak normalized to 1.0) using single radionuclide obtained from a myocardial phantom. In addition, the energy spectra for various ^99m^Tc/^123^I count ratio were obtained from mixed radionuclide phantom acquisition and normalized by the peak total count of ^123^I. We confirmed that the experimentally acquired spectra closely matched the composite spectra generated from the individual radionuclides, demonstrating the validity of spectral superposition for modeling dual-isotope acquisition (Fig. 2b). The energy values were respectively set at 105‒130 keV and 133‒145 keV for the ^99m^Tc scatter and main windows, and at 145‒152 keV, 152‒168 keV, and 173‒196 keV for the ^123^I scatter, main, and upper windows, respectively. The relative counts and ratios of ^99m^Tc and ^123^I were calculated by the energy spectra of ^123^I and ^99m^Tc single radionuclide conditions using Wolfram Language (Ver. 14.2, Wolfram Research, Inc, Champaign, IL, USA). The contamination ratio of ^123^I in the ^99m^Tc main window was calculated by the following Eq. 1:

1 $$\:Contamination\:ratio=\:\frac{{123}_{I}\:total\:counts}{{99m}_{Tc\:total\:counts}}$$

Short-axis images were reconstructed without (NC) and with (SC) scatter correction using a proprietary algorithm (Spectrum Dynamics Medical Ltd.) (Gambhir et al. 2009). Subsets and iterations were 32 and 4, respectively. The default kernel and Gaussian filter standard were 1 and 1.0, respectively, and we modified them to 1, 3 and 5, and 0.25–1.0 using Eqs. 2 and 3, respectively.

2 $$\:G(x,\:y,\:z)=\:\frac{1}{{\left({2\pi\:\sigma\:}^{2}\right)}^{3/2}}exp\left(-\frac{{x}^{2}+{y}^{2}+{z}^{2}}{{2\sigma\:}^{2}}\right)$$

3 $$\:Kernel\hspace{0.17em}=\hspace{0.17em}2\times\:3\sigma\hspace{0.17em}+\hspace{0.17em}1$$

where x, y, and z indicate spatial coordinates, and σ represents the Gaussian filter standard. Scatter was corrected using a proprietary method (Spectrum Dynamics Medical Ltd.) (Holstensson et al. 2015).

The percent coefficient of variance (%CV) was calculated using each %uptake from a 17-segment polar map of a normal myocardium. Cavity contrast was calculated using a square region of interest (ROI) on the LV cavity and the anterior myocardial wall in short-axis images. The average %uptake of defective areas (segment numbers 4 and 10; mid and basal inferior segments) was also obtained from a 17-segment polar map.

We evaluated the effects of various filter applications on ^99m^Tc images with NC and SC under clinical conditions. Clinical samples at 7 days after coronary revascularization were assessed by SDI acquisition using ^99m^Tc-tetrofosmin (370 MBq) and ^123^I-BMIPP (111 MBq). The Ethics Committee at Kanazawa University approved the study protocol (Approval ID: 2019 − 244 (3266)).

Data were statistically analyzed using EZR (Saitama Medical Center, Jichi Medical University, Saitama, Japan), which has a graphic user interface for R version 3.3.2 (The R Foundation for Statistical Computing, Vienna, Austria) (Kanda 2013). The differences of %uptake for normal myocardial polar map between NC and SC images were analyzed by paired t-test. In addition, ^99m^Tc/^123^I ratio was used by Kruskal–Wallis tests, and post hoc analyses comprised Steal-Dwass tests. All statistical tests were two-tailed, and values with *p* < 0.05 were considered significant.

## Results

Figure 2b represents the energy spectra of the myocardial phantom for different ^99m^Tc/^123^I count ratios. The fractions of ^123^I in the total counts within the ^99m^Tc window were 59%, 32%, 22%, and 18% for the ^99m^Tc/^123^I ratio of 1.0, 3.5, 5.0 and 6.5, respectively (Fig. 2c). Correspondingly, the ratios of ^123^I to ^99m^Tc counts within the ^99m^Tc window were 1.44, 0.48. 0.29, and 0.22 (Fig. 2d).

**Fig. 3:**
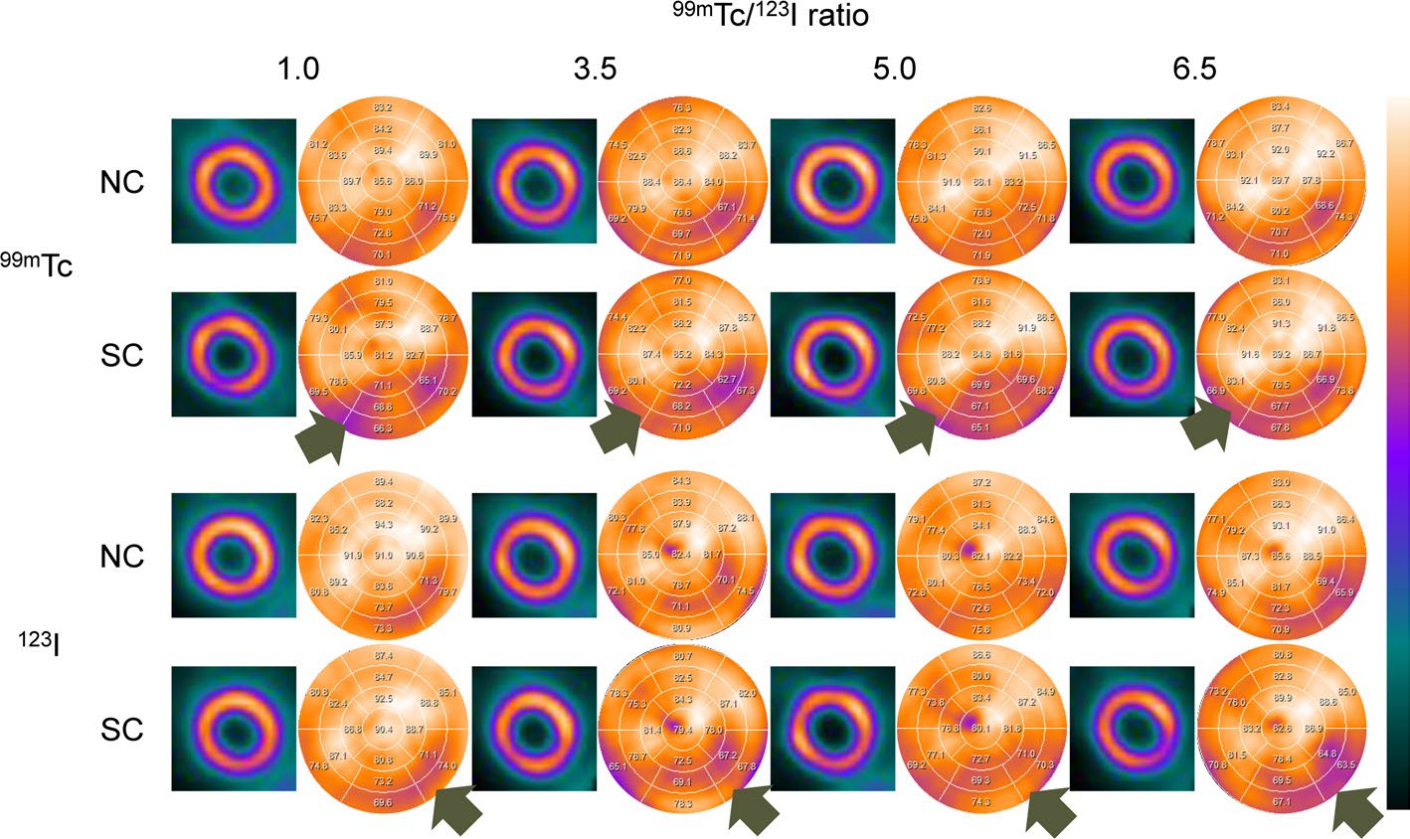
Visual and quantitative evaluation of normal myocardial phantom. Gaussian standard and kernel values were both set to 1

**Fig. 4:**
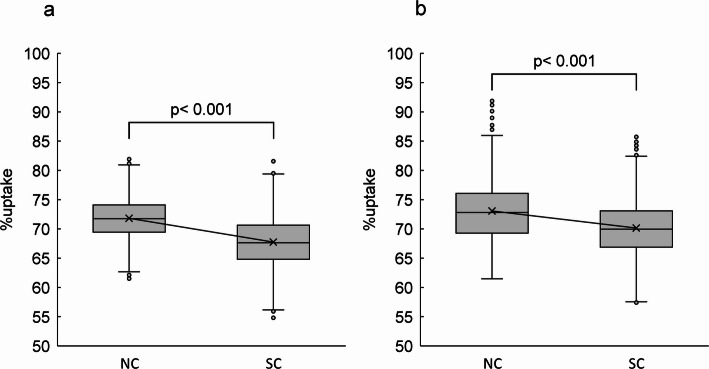
The %uptake of ^99m^Tc (**a**) and ^123^I (**b**) for the inferior and inferolateral myocardial walls (Segments 4, 5, 10 and 11) in the normal myocardium. NC, no correction; SC, scatter correction

Normal myocardial images and polar maps for each of ^99m^Tc and ^123^I under default Gaussian filter conditions are shown in Fig. 3. In the normal myocardium, the average %uptake in the inferior and inferolateral myocardial walls (Segments 4, 5, 10 and 11) was respectively 71.8 ± 3.9%, 67.8 ± 4.7%, 73.1% ± 5.4%, and 70.2 ± 5.3% for ^99m^Tc NC, ^99m^Tc SC, ^123^I NC and ^123^I SC. The average %uptake in SC images was significantly decreased compared to NC images for both radionuclides (Fig. 4). The average %uptake of ^99m^Tc SC image in the inferior and inferoseptal myocardial walls (Segments 3, 4, 9 and 10) of a normal myocardium was 72.9 ± 9.3%, 72.7 ± 8.4%, 70.4 ± 6.3%, and 69.5 ± 8.2% for Tc/I_6.5, Tc/I_5.0, Tc/I_3.5, and Tc/I_1.0, respectively. In addition, the average %uptake of ^123^I SC image in the inferior and inferolateral myocardial walls (Segments 4, 5, 10 and 11) of a normal myocardium was 66.1 ± 4.7%, 71.1 ± 3.5%, 70.7 ± 5.2%, and 72.8 ± 5.1% for Tc/I_6.5, Tc/I_5.0, Tc/I_3.5, and Tc/I_1.0, respectively.

**Fig. 5:**
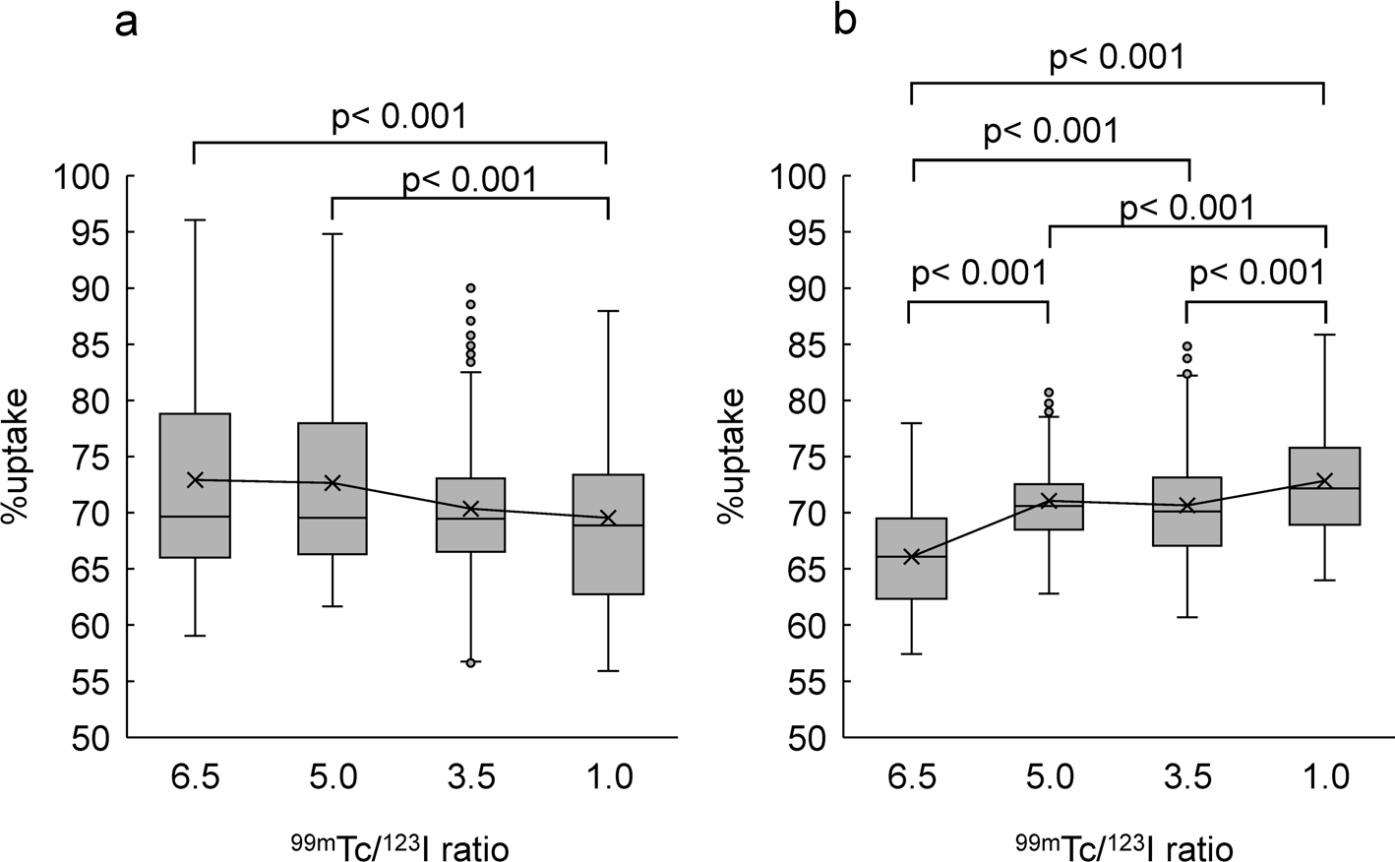
The %uptake of normal myocardium according to the ^99m^Tc/^123^I ratio. In the scatter-corrected (SC) images, ^99m^Tc showed %uptake in the inferior and inferoseptal myocardial walls (Segments 3, 4, 9, and 10), whereas ^123^I showed %uptake in the inferior and inferolateral myocardial walls (Segments 4, 5, 10, and 11)

As illustrated in Fig. 5, ^99m^Tc SC images with Tc/I_1.0 and Tc/I_3.5 showed significantly decreased counts in the inferoseptal wall compared with other ^99m^Tc/^123^I ratios. Additionally, ^123^I SC images with Tc/I_6.5 demonstrated significantly lower counts compared with other ratios.

**Fig. 6:**
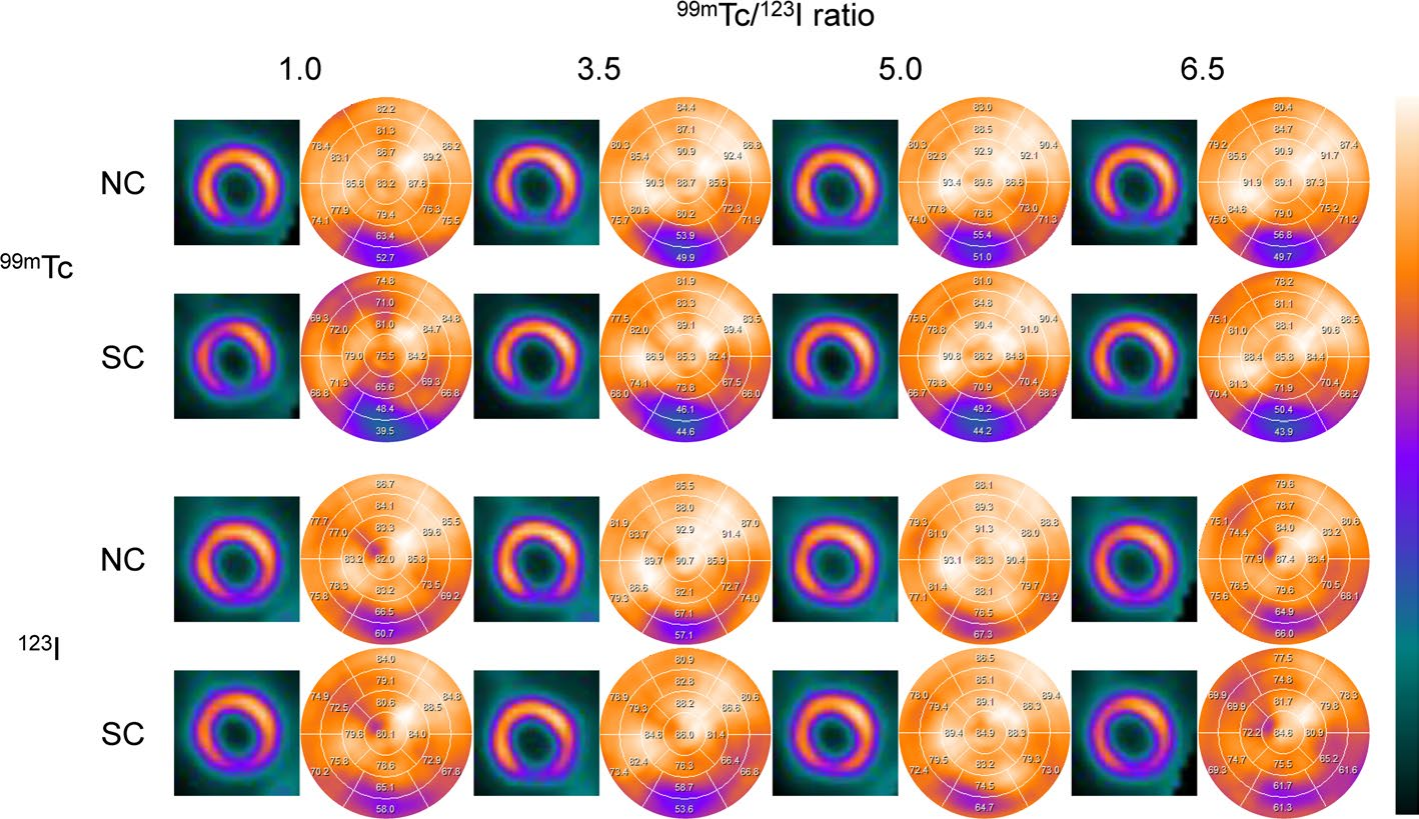
Visual and quantitative evaluation of myocardium with inferior defect. Bottom of short axis image shows minimum %uptake by defect in circumferential profile curve

Myocardial images of an inferior defect and polar maps assessed by ^99m^Tc and ^123^I under default Gaussian filter conditions are shown in Fig. 6. The average %uptake in the defect region (Segments 4 and 10) on ^99m^Tc images for Tc/I_1.0, Tc/I_3.5, Tc/I_5.0 and Tc/I_6.5 was 57.3 ± 8.8%, 51.3 ± 8.4%, 53.1 ± 9.0% and 52.2 ± 8.1% for NC, and 43.9 ± 10.6%, 45.6 ± 7.7%, 46.1 ± 9.3% and 45.8 ± 7.4% for SC, respectively. The % uptake of the defect on ^99m^Tc NC image was significantly higher at Tc/I_1.0 compared with other ^99m^Tc/^123^I ratios (*p* < 0.001). The %uptake of the defect in Tc/I_1.0 SC images was significantly decreased compared with other ^99m^Tc/^123^I ratios (*p* < 0.001). For ^123^I images the % uptake in the defect region was 60.2% to 71.8% for NC and 56.0% to 69.7% for SC, respectively. The defect detectability was significantly lower in ^123^I NC images than in ^99m^Tc NC images (*p* < 0.001), and application of SC slightly improved the %uptake in ^123^I NC image (*p* < 0.001).

**Fig. 7:**
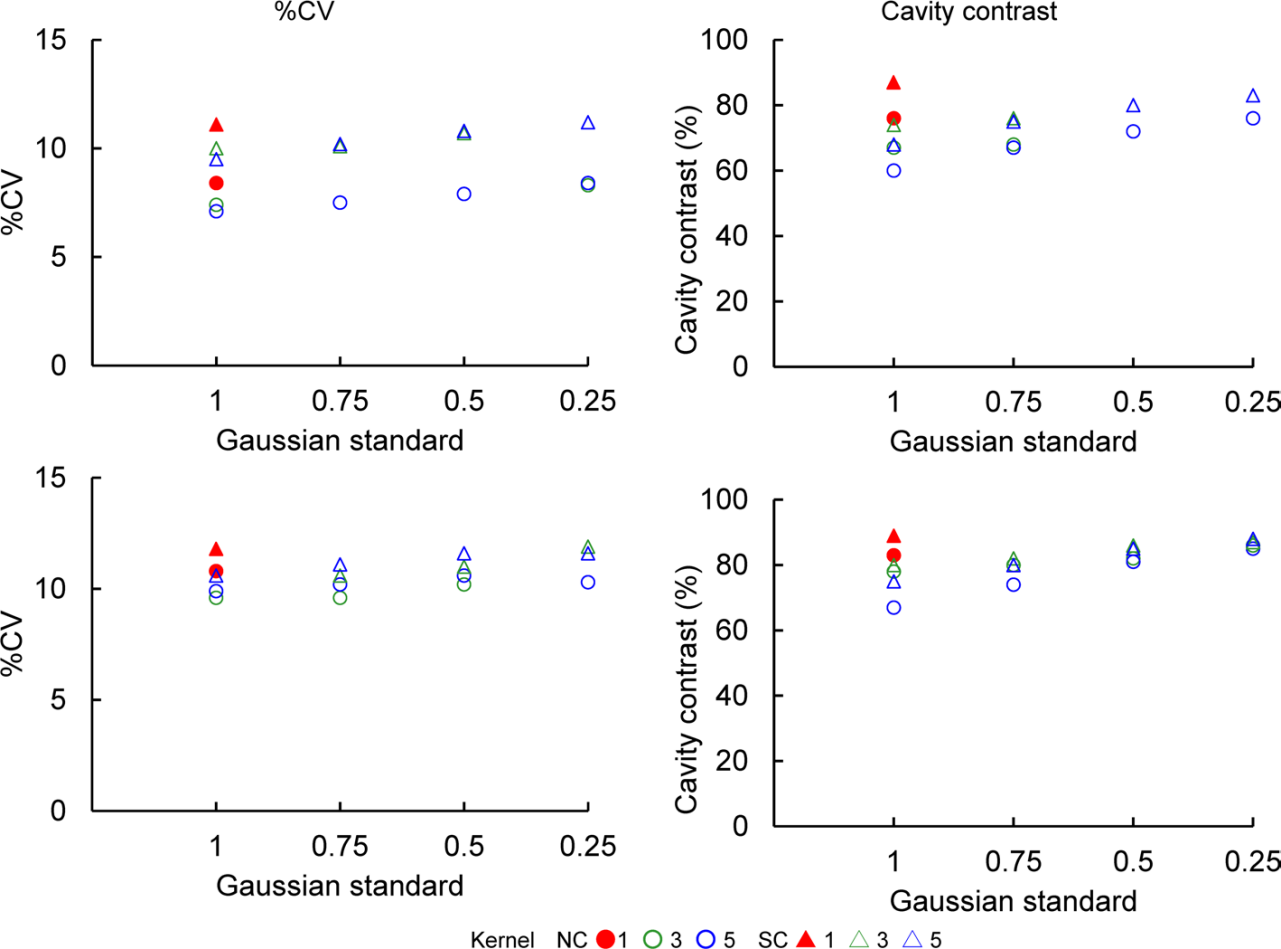
Comparison of %CV and cavity contrast with various standards and kernels of Gaussian filter in normal myocardium. Left and right panels: %CV and cavity contrast, respectively. Upper and lower panels: ^99m^Tc image with ^99m^Tc/^123^I 1.0 and ^123^I image with ^99m^Tc/^123^I 6.5, respectively. %CV, percent coefficient of variance; Std, standard, ^99m^Tc/^123^I, Technetium-99m to Iodine-123 ratio

Figure 7 shows the %CV and cavity contrast of images with ^99m^Tc under Tc/I_1.0, and with ^123^I under Tc/I_6.5. In both NC and SC images for ^99m^Tc and ^123^I, %CV decreased with increasing kernel size and Gaussian standard values. Under Gaussian standards of 0.25–0.75, %CV remained relatively stable regardless of the kernel size. However, at a Gaussian standard of 1.0, the kernel had a more noticeable influence. Across all conditions, NC images consistently exhibited lower %CVs than SC images. Cavity contrast also declined in NC and SC images as kernel size and Gaussian standard values increased. While cavity contrast was generally unaffected by the kernel size at Gaussian standards of 0.25–0.75, a kernel-dependent reduction was observed Gaussian standard of 1.0, mirroring the trends seen for %CV. In addition, cavity contrast decreased as kernel values increased in the NC and SC images when the Gaussian standard was 1.0.

**Fig. 8:**
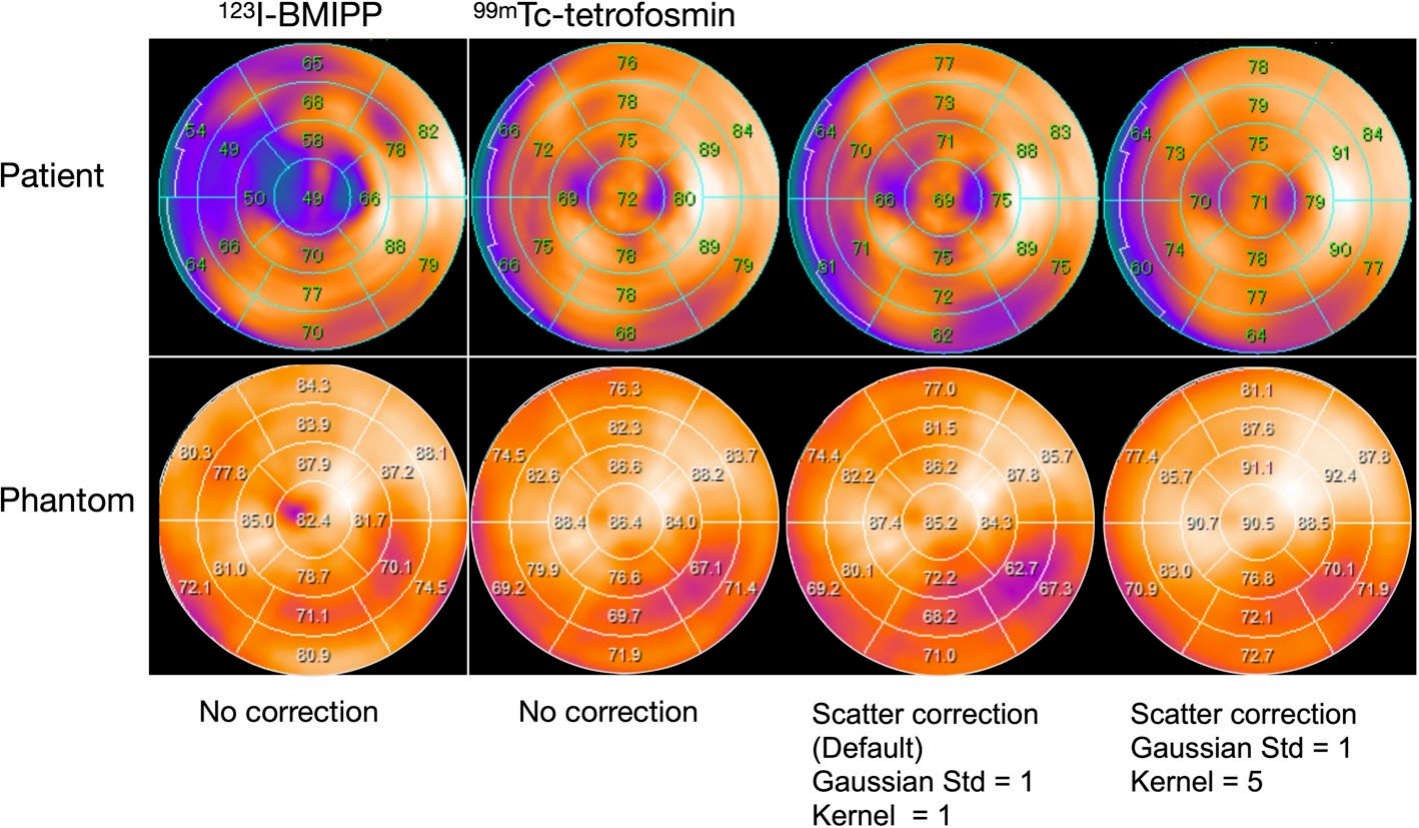
Polar maps of a clinical case (upper panel) and corresponding phantom study (lower panel) under matched image processing conditions. The upper panel shows the polar map from a patient with acute coronary syndrome (an anteroseptal infarction, treated by percutaneous coronary intervention). The lower panel displays the polar map from a normal myocardial phantom imaged at a ^99m^Tc/^123^I ratio of 3.5 (Tc/I_3.5), under corresponding acquisition settings. Tc/I, ^99m^Tc to ^123^I ratio. The SC image of kernel = 5 for ^99m^Tc polar map was improved the decrease in inferolateral wall accumulation compared with the default setting of ^99m^Tc SC image

Polar maps derived from an 82-year-old male patient and normal myocardial phantom with Tc/I_3.5 are shown under comparable conditions (Fig. 8). This patient diagnosed with ACS was treated by percutaneous coronary intervention (PCI) in the left anterior descending artery (coronary segments 6 and 7). During simultaneous dual-isotope acquisition, the myocardial count ratio between ^99m^Tc-tetrofosmin and ^123^I-BMIPP in the myocardium was directly measured and found to be 1.15. An obvious anteroseptal mismatch between ^123^I-BMIPP and ^99m^Tc-tetrofosmin reflected ischemic memory. While the activity in the inferolateral region was slightly reduced at the default SC setting (Gaussian standard, 1; kernel, 1), increasing to kernel 5 improved %uptake in this area, rendering it comparable to that observed in the NC image.

## Discussion

Myocardial SDI imaging is a valuable imaging technique that enables the acquisition of multiple molecular imaging datasets within the same session, allowing simultaneous physiological assessment (Du et al. 2013, 2014). However, SDI using ^99m^Tc and ^123^I on a CZT cardiac camera has certain limitations, including crosstalk and downscatter, which can impair quantitative accuracy and image contrast (Niimi et al. 2018; Blaire et al. 2018). In conventional NaI-based SPECT cameras, such artifacts are commonly addressed using energy window-based methods such as triple-energy window (TEW) correction However, a CZT detector generates a tailing effect on the low-energy side of the photopeak even without patient scatter. This is attributed to incomplete charge collection and inter-crystal scattering within the detector, which leads to enhanced crosstalk of low-energy radionuclides in SDI acquisition. As a result, TEW-based SC can lead to overestimation (Pourmoghaddas et al. 2015). To address these issues, the D-SPECT CZT camera employs a proprietary SC algorithm (Gambhir et al. 2009). However, the fixed nature of its correction parameters raises uncertainty about its robustness across varying ^99m^Tc/^123^I ratios.

Although various ^99m^Tc/^123^I ratios have been applied in previous studies (Niimi et al. 2018; Assante et al. 2022; Yamada et al. 2021; Blaire et al. 2016, 2018; Takahashi et al. 2015), their impact on the ^99m^Tc/^123^I ratio has not been systematically investigated. The degree of ^123^I contamination within the ^99m^Tc energy window was found to vary markedly with changes in the^99m^Tc/^123^I activity ratios. Specifically, the image quality of ^99m^Tc with SDI is affected by Compton scatter from ^123^I, whereas that of ^123^I with SDI is affected by crosstalk. In addition, the level of scattered radiation in the myocardium is particularly influenced in the adjacent inferior wall due to extracardiac activity, such as that originating from the liver and gallbladder. Consequently, SC produces the most pronounced changes in the inferior wall of the myocardium (Harel et al. 2001). Therefore, we focused our analysis on the myocardial distribution in the inferior wall.

The photopeak count was lower for ^99m^Tc than for ^123^I under Tc/I_1.0. Although the energy spectrum of ^123^I was consistent due to acquisition in a preset count mode, Compton scatter from ^123^I similarly contributed to the ^99m^Tc spectrum across all ^99m^Tc/^123^I ratio. Furthermore, SC using energy windows similar to multiple energy window method (Torizuka et al. 1991) removes ^99m^Tc signals along with scattered ^123^I rays. Consequently, the ^99m^Tc SC image of Tc/I_1.0 was heterogeneous in the inferior wall of the normal myocardium due to insufficient counts.

The ^123^I SC image at Tc/I_6.5 revealed heterogeneous distribution in the normal myocardium with reduced activity in the inferolateral wall. The CZT camera has better energy resolution than conventional NaI(Tl) detectors and the images are less affected by crosstalk (Torizuka et al. 1991). However, crosstalk was not fully eliminated (Fig. 2). Myocardial images of ^123^I with higher ^99m^Tc/^123^I ratios were more susceptible to crosstalk, which contributed to the heterogeneity in images of the normal myocardium with Tc/I_6.5. In addition, the ^123^I image quality might be affected by septal penetration by 529 keV γ rays (Inoue et al. 2004; Chen et al. 2006). The septum thickness and length of the tungsten collimator of D-SPECT are 0.2 and 21.7 mm, respectively, whereas those of the LEHR collimator are 0.15‒0.20 and 24–35 mm, respectively. Tungsten collimators provide a higher shielding capacity than conventional lead collimators, The reported performance of the ^123^I for tungsten collimator D-SPECT is 5%–6%. This has a higher penetration ratio than ^123^I-specific low-medium energy general-purpose, low-penetration high-resolution, and expended low-energy general-purpose collimators (Sato and Hayashi 2020). Since the LV ^123^I count was set to 1.5 × 10^6^ at all ^99m^Tc/^123^I ratios, the effect of septal penetration on image quality was unlikely to vary among ^99m^Tc/^123^I ratios. Therefore, the heterogeneous distribution of ^123^I at Tc/I_6.5 in the normal myocardium was probably due to crosstalk from ^99m^Tc.

Since the SC parameters are device-specific, we proposed adjusting the Gaussian filter parameter to improve the heterogeneous distribution observed the inferior wall of the normal myocardium. The kernel of a Gaussian filter determines the spatial extent of smoothing; a larger kernels corresponds to a higher Gaussian standard value, resulting in a stronger smoothing effect. When the kernel is set to 1, the Gaussian standard is internally fixed at 1, representing minimal smoothing. The %CV of ^99m^Tc at Tc/I_1.0 and of ^123^I at Tc/I_6.5 in SC images was slightly improved by increasing the kernel value of the Gaussian filter from the default setting. Furthermore, NC images enhanced image uniformity while preserving defect detectability. When the ^99m^Tc/^123^I ratio is particularly high or low, adjusting the kernel parameter of the Gaussian filter or simply using NC images might improve the quality. In general, myocardial imaging with SC without attenuation correction (AC) can result in heterogeneous distribution (Zoccarato et al. 2014). Because our D-SPECT scanner does not include computed tomography-based AC module, AC could not be applied. Therefore, NC images may represent a viable clinical alternative under such circumstances.

The clinical application of our phantom findings appears promising. In our initial clinical case involving a patient after PCI, the results closely mirrored those observed in the phantom study, as shown in Fig. 8. By adjusting the kernel parameter of the Gaussian filter, we improved homogeneity of ^99m^Tc SC polar map, including relatively decreased inferolateral wall counts. The broad clinical application of dual nuclide ^123^I-BMIPP and ^99m^Tc-perfusion studies requires validation in patients with ACS, complications of ischemia and infarction, vasospastic angina, and Takotsubo syndrome. The impact of these corrections on diagnostic accuracy and prognostic assessment will be essential to evaluate given the various defect and mismatch scoring methods in clinical practice. The ability of ^123^I-BMIPP to diagnose coronary artery disease must be proven as it is underutilized outside Japan (Otaki 2024; Nakajima et al. 2020).

Spectrum Dynamics Medical has developed a new AC map using a deep learning algorithm (Ochoa-Figueroa et al. 2024). Although we could not correct attenuation in the present study, we believe that it has potential to mitigate the heterogeneity introduced by SC. Previous studies have demonstrated that combining AC and SC enables more accurate myocardial activity distribution (Harel et al. 2001; Banzo et al. 2003; Ohyama et al. 2001; Okuda et al. 2009). Further studies should evaluate SDI imaging with ^99m^Tc and ^123^I incorporating AC to further improve image quality and diagnostic reliablity.

## Conclusion

We assessed image quality and the effects of SC on ^99m^Tc and ^123^I images across different ^99m^Tc/^123^I ratios using the D-SPECT Cardio CZT camera. The ^123^I SC image at Tc/I_6.5 and the ^99m^Tc SC image at Tc/I_1.0 revealed heterogeneity in the inferior wall of the normal myocardium. These findings may be attributed to overcorrection effects introduced by the proprietary SC algorithm. When the ^99m^Tc/^123^I ratio is particularly high or low, image quality might be improved by adjusting the kernel parameter of Gaussian filter. Alternatively, images without SC may provide clinically acceptable results, especially in the system lacking AC capability. Further optimization and validation, particularly incorporating AC, are needed for broader clinical application of SDI.

## Data Availability

Without authorization from the Ethics Committees of Kanazawa University, the image datasets generated or analyzed in this research are not disclosed publicly. However, the corresponding author may provide them upon reasonable request.

## Abbreviations

AC: Attenuation correction
CV: Coefficient of variance
CZT: Cadmium-zinc-telluride
LV: Left ventricular
NC: No correction (without scatter correction)
SC: Scatter correction
SDI: Simultaneous dual-isotope
SPECT: Single photon emission computed tomography
